# Parental influences on child physical activity and screen viewing time: a population based study

**DOI:** 10.1186/1471-2458-10-593

**Published:** 2010-10-08

**Authors:** Ben J Smith, Anne Grunseit, Louise L Hardy, Lesley King, Luke Wolfenden, Andrew Milat

**Affiliations:** 1 Department of Health Social Science, Monash University, PO Box 197, Caulfield East, Melbourne, Victoria, 3145, Australia; 2 Physical Activity Nutrition Obesity Research Group, University of Sydney, Sydney, New South Wales, Australia; 3 Hunter New England Population Health and School of Medicine and Public Health, University of Newcastle, Callaghan, New South Wales, Australia; 4 New South Wales Health Department, North Sydney, New South Wales, Australia

## Abstract

**Background:**

Parents can influence their children's physical activity participation and screen time.This study examined the relative significance of perceived parental barriers and self-efficacy in relation to children's physical activity participation and screen time viewing. The associations between these factors and the behaviours were analysed.

**Methods:**

Cross-sectional population survey in New South Wales, Australia of parents of pre-school (N = 764), younger (Kindergarten, Grades 2 and 4; N = 1557) and older children (Grades 6, 8 and 10; N = 1665). Parents reported barriers and self-efficacy to influence their child's physical activity and screen time behaviours in a range of circumstances. Differences were examined by child's sex and age group, household income, maternal education and location of residence. The duration of physical activity and screen viewing was measured by parental report for pre-school and younger children and self-report for older children. Associations between parental factors and children's organised, non-organised and total activity and screen time were analysed.

**Results:**

Cost, lack of opportunities for participation and transport problems were the barriers most often reported, particularly by low income parents and those in rural areas. The number of barriers was inversely related to children's time spent in organised activity, but not their non-organised activity. Higher parental self-efficacy was positively associated with organised physical activity in the younger and older children's groups and the non-organised activity of older children. School-age children (younger and older groups) were less likely to meet physical activity guidelines when parents reported ≥4 barriers (OR 3.76, 95% CI 1.25-11.34 and OR 3.72, 95% CI 1.71-8.11 respectively). Low parental self-efficacy was also associated with the likelihood of children exceeding screen time guidelines for each age group (pre-school OR 0.62, 95% CI 0.43-0.87; young children OR 0.56, 95% CI 0.39-0.80; and older children OR 0.57, 95% CI 0.43-0.74).

**Conclusion:**

Parental barriers are associated with the time that children spend in both active and sedentary pursuits. These findings highlight family, economic and environmental factors that should be addressed in programs to promote child physical activity and tackle sedentary behaviour.

## Background

The rising prevalence of overweight and obesity among children is a public health issue of global importance [1,2]. Population studies conducted in Australia, Canada, Ireland, Italy, Malta, Mexico, Slovenia and the United States have classified over a quarter of those measured across the 5-18 year age range as overweight or obese [3]. Alongside these epidemiological studies has been a large body of research examining the determinants of unhealthy weight gain. High intake of energy dense foods [4], low levels of physical activity [5] and prolonged screen viewing [6] have been identified as causal factors with the potential to be modified. This body of research has contributed to an understanding of the lifestyle patterns that are developed during youth, which may be carried into adulthood, and the risk of early chronic disease development [7]

The development of effective policies and programs to increase the time children spend in physical activity on a regular basis, and to reduce television viewing and other modifiable sedentary pursuits, requires an analysis of the psychological, social, environmental and economic factors that influence these behaviours. Studies among children and youth have investigated the causes and correlates of total physical activity time [8,9], participation in sports and organised activities [10] and time spent in sedentary pursuits [11]. The family environment has been consistently identified as a significant influence on both physical activity and dietary intake [12,13].

It has been reported that the level of support offered by parents and whether they are role models by their own levels of participation, can influence the time children spend in physical activity or screen viewing [14]. Parents may determine children's exposure to a number of factors that are enablers or barriers to physical activity, such as money to finance the costs of participation, sports and exercise equipment, transportation to attend activities, and availability of television and other screen activities [9,14,15]. Consequently, given parents' central role, a number of interventions addressing physical activity, sedentariness and overweight and obesity in children have sought to engage parents intensively [13,16].

While there is clear evidence about the significance of parental influence, there has been limited analysis of the factors which affect the nature and extent of this influence on child behaviours, including psychosocial, environmental, geographic and economic factors. Further, few studies have examined how parents' influences vary according to children's age. The aim of this study was to investigate how parental self-efficacy and perceived barriers are associated with children's physical activity and screen viewing time. Secondly, the study examined how these relationships differ according to children's age, and household socio-economic and demographic characteristics.

## Methods

### Design and setting

A cross-sectional survey of children attending preschool and long day care centres, primary (Kindergarten, Grades 2, 4, 6) and high school (Grades 8 and 10) was conducted in the Hunter New England region of New South Wales (NSW) Australia. The study was approved by the Hunter New England Area Health Ethics Committee, the NSW Department of Education and Training and the NSW Catholic Education Commission and data were collected between February and September 2007.

### Participants

Parents of children from 16 preschools and 24 long day care centres, randomly selected from a list of licensed childcare centers were invited to participate. Child care centres with < 20 children and those catering for children with special needs (e.g., autism, blindness) were excluded. Thirty-five primary and 35 high schools in total were also randomly selected from each education sector (Government, Catholic and Independent) proportional to the number of students enrolled in that sector. Schools which catered for children with special needs and in remote locations were excluded. Within each school, two classes were randomly chosen from each of the Grades being surveyed and all students invited to participate. Written consent by children and their carers was a requirement for participation.

### Questionnaire items

The survey completed by all parents measured perceived barriers and self-efficacy to influence their child's physical activity participation. The physical activity and screen viewing time of preschool and younger children was reported by parents, whereas older children self-completed these measures in a separate survey. Demographic information collected from parents included the child's sex, date of birth, school year, residential postcode, household income and maternal education. Household income was chosen as an indicator of socioeconomic status (SES) [17] while residential postcode was used to classify children living in an 'urban' or 'rural' locality.

### Barriers and self-efficacy

Parents were asked to what extent they agreed with five statements that described barriers which restricted their child's participation in physical activity. These were derived from the parent survey used to evaluate the *VERB *child physical activity campaign in the United States, in which they were found to have acceptable item-response and good test-retest reliability [18]. The statements referred to issues concerning availability of transportation, opportunities for activities in the vicinity of their home, the cost of activities, time constraints of parents, and availability of activities that the child likes. For each statement parents were asked to rate their agreement on a 5-point Likert scale (strongly agree to strongly disagree).

For self efficacy parents were asked to rate their confidence to influence their child's physical activity in a series of challenging situations, which were identified from focus group consultations with parents undertaken by the NSW Health Department (unpublished document). The following eight situations were included: parent does not have much time; the child is engaged in screen time activities; the parent feels stressed; the child does not have a friend to play with; the parent cannot think of activities to suggest; the child is not interested; the parent is not able to participate in the activity, and; the child's preferred activity is expensive. Parents were asked to indicate whether they were very confident, confident, a little confident or not confident in each of these circumstances. Factor analysis showed only one underlying factor for this set of questions in the study sample with good internal reliability (Cronbach's Alpha = 0.88).

### Physical activity and screen viewing time

The physical activity questions measured the usual time each spent in organised and non-organised activities on a daily basis while the screen viewing questions measured the usual time spent in a range of screen activities (i.e., TV, recreational computer use, electronic games) each day. The parent report measures used for preschool and younger children were drawn from the NSW Population Health Survey [19] and were adopted based on research supporting the accuracy of parent proxy reports of physical activity among children under 12 years [20]. The self-report physical activity questions used with older children have been reported to have acceptable retest-reliability and validity (compared with fitness tests) among 13-15 year olds [21]. Previous evaluation of the screen viewing questions completed by older children has shown these measures to have very good re-test reliability in 11-15 year olds [22].

### Statistical analysis

Separate analyses were undertaken for preschoolers (attending preschool or long day care centres), younger (school grades K, 2, and 4) and older children (school grades 6, 8, and 10) Demographic covariates were: child's sex; maternal education, categorised as lower (not completing high school), intermediate (completing high school or a vocational diploma), or higher (completing a university degree); residential locality (urban or rural), and; household income (< $40,000, ≥$40,000-$100,000 or ≥$100,000).

For questions about barriers to physical activity the "strongly agree" and "agree" responses were combined, as were "strongly disagree" and "disagree", to create binary variables for each item. The neutral option was collapsed with the answer option that indicated that the respondent did not report a barrier for that item. A 'total barriers' score was derived by summing the number of barriers reported.

Similarly, response options for "not confident" and "a little confident" were combined, as were those for "confident" and "very confident" to form binary outcome variables for the analysis of individual self-efficacy questions. A self-efficacy to influence physical activity scale score was also created from parent responses on the eight questions. Higher scores represented greater self-efficacy among parents to influence their child's physical activity. Because scores on the scale were highly skewed, a categorical variable was then derived splitting the sample into three groups; scores ≤ 25^th ^percentile on the scale were assigned to the low self-efficacy group, scores >25^th ^but <75^th ^percentile were in the moderate self-efficacy group and those with scores ≥ 75^th ^percentile were assigned to the high self-efficacy group. The distribution of parents across these three groups was approximately equal.

A categorical variable for physical activity participation was generated because the data were not normally distributed. Both organised and non-organised activity were categorised to reflect no activity (0 minutes/day), low to medium activity (1-59 mins/day) and high activity (≥ 60 mins/day). Children were also classified according to whether the sum of their organised and non-organised daily activity equalled the physical activity guidelines issued for children, which are ≥ 60 mins/day for younger and older children [23] and ≥ 3 hours per day for preschoolers [24]. Screen viewing time was summed and categorised according to guidelines (i.e., <2 or ≥2 hrs/day) [25].

Bivariate analyses were conducted using Chi-square while logistic regression methods were used in multiple variable analyses. All analyses were adjusted for clustering within school using STATA version 10.0 complex survey commands. The significance level was set at 5%, however, multiple comparisons within significant multi-category independent variables were Bonferonni adjusted for the number of comparisons to reduce the likelihood of Type I error.

## Results

### Sample characteristics

The sample comprised 4006 children for whom parent surveys were also completed: a preschool group (n = 764); a younger children's group (n = 1557); and an older children's group (n = 1685). The mean ages for each group were 3.9 years (SD 0.8), 7.6 (SD 1.7) years and 13.6 (SD 1.5) years respectively. The proportion of children from participating schools and child care centres for whom consent was provided and complete data were obtained for both parents and children was 55%. The demographic characteristics of the children are shown in Table 1.

**Table 1:** Demographic characteristics of the sample

Characteristic	Preschool group	Younger group†	Older group‡
**n**	764	1,557	1,685
**Boys (%)**	50.3	48.6	47.9
**Mean age in years (range)** **Std Dev**	3.9 (1.7-5.6) SD 0.8	7.6 (4.3-13.6) SD 1.7	13.6 (9.9-17.0) SD 1.5
**Household income (%)**			
< $40,000	28.4	28.9	24.3
$40,000-$100,00	52.0	53.3	53.2
> $100,000	19.7	17.8	22.5
Maternal education (%)*			
Lower	23.2	33.1	35.6
Intermediate	11.4	13.1	10.7
Higher	65.4	53.8	53.7
**Locality (%)**			
Urban	47.8	45.7	43.8
Rural	52.2	54.3	56.2

† Grades K, 2 & 4

‡ Grades 6, 8 & 10

* Lower-not completed high school; Intermediate-completed high school or vocational diploma; Higher - completed university degree

### Parent reported barriers and self-efficacy

Table 2 shows that the most frequent barrier reported by parents was the cost of activities. This was followed by lack of physical activity opportunities, time constraints and transportation which were each reported by approximately one-fifth of parents. Household income was consistently associated with all barriers except lack of time. The prevalence of cost as a barrier also differed with the child's age group and level of maternal education (*p *< .01). Overall, almost a third (30.4%) of parents reported two or more barriers to facilitating their child's physical activity. There were significant differences in the number of barriers reported according to the child's age group (*p *= .04), household income (*p *< .01) and location of residence (*p *< .01).

**Table 2:** Adjusted percentage of parents reporting barriers to facilitating physical activity by child age group and demographic characteristics

*Barriers*	Total	Age group				Household income				Maternal education				Locality		
		Pre-school	Young	Older	p*	< 40 k	40- < 100 k	100 k+	p*	Lower	Intermediate	Higher	p*	Urban	Rural	p*
Issues with transportation	17.5	9.2	18.8	17.5	.12	26.6	15.3	9.6	< .01	19.2	15.0	15.6	.23	11.4	22.2	< .01
Lack of opportunities for physical activity	21.3	27.3	23.7	20.1	.29	29.3	20.5	12.7	< .01	23.7	20.2	17.7	.17	12.2	28.5	< .01
Some activities too expensive	37.9	37.2	46.4	34.3	< .01	55.9	38.3	14.3	< .01	42.5	39.3	28.3	< .01	38.1	37.7	.91
Insufficient time	18.5	22.7	23.5	16.1	< .01	19.1	19.2	16.9	.59	16.4	17.7	20.6	.21	16.4	20.1	.05
Child doesn't like physical activity	12.8	4.9	13.1	13.2	.06	16.7	11.6	8.7	< .01	15.6	10.9	9.8	< .01	12.4	13.1	.74

Total barriers to physical activity																
0-1	69.5	72.4	64.9	71.3	.04	55.6	71.7	83.7	< .01	65.9	71.5	74.8	.06	76.6	64.0	< .01
2-3	26.0	25.9	30.6	24.1		36.8	23.7	15.7		29.6	23.9	22.9		20.1	30.6	
≥ 4	4.4	1.7	4.5	4.6		7.7	4.7	0.6		4.5	4.6	2.3		3.3	5.4	

*Significance of overall Chi-square test of association

Table 3 shows that the level of parental self-efficacy to influence their child's physical activity participation differed significantly by the child's age group (*p *< .01) and household income (*p *= .01). The most common contexts in which parents reported lower self-efficacy were when the child was not interested, when they considered the activity to be expensive, when they were feeling stressed and when they were unable to suggest an activity for the child to undertake. The proportion of parents reporting low self-efficacy varied by household income for some circumstances (when they considered the activity expensive, when they felt stressed and their child had no friend to play with); and by maternal education in certain circumstances (when the activity was expensive, when they were unable to suggest an activity and the parents were unable to join in).

**Table 3:** Adjusted percentage of parents reporting low self-efficacy to influence physical activity by child age group and demographic characteristics

*Reason for low* *self-efficacy*	Total	Age group				Household income				Maternal education				Locality		
		Pre-school	Young	Older	p*	< 40 k	40- < 100 k	100 k+	p*	Lower	Intermediate	Higher	p*	Urban	Rural	p*
Not much time	34.3	38.7	33.5	34.3	.47	35.8	33.6	33.5	.74	35.1	31.4	37.8	.12	34.9	33.9	.71
Child watching TV	27.3	14.0	18.9	31.7	< .01	27.6	27.2	28.4	.93	30.3	23.9	28.4	.08	27.8	27.0	.74
Feeling stressed	41.6	42.9	38.7	42.7	.10	46.5	40.7	37.9	.03	41.3	41.3	42.8	.84	41.2	41.9	.77
Child has no friend to play with	29.6	15.5	25.1	32.5	< .01	33.2	30.2	24.3	.03	32.9	29.1	27.6	.06	32.0	27.9	.06
Can't suggest activity	39.4	31.1	33.8	42.3	< .01	38.5	39.2	38.8	.98	43.3	36.9	38.6	.04	40.7	38.5	.33
Child not interested	51.9	44.4	47.8	54.2	< .01	53.8	52.1	50.6	.69	51.1	53.6	51.5	.64	53.5	51.0	.32
Can't join in	30.7	31.3	27.2	32.1	.03	30.8	30.9	27.1	.41	34.7	28.1	27.9	.03	33.0	28.9	.09
Activity too expensive	44.5	44.2	45.7	44.1	.64	57.6	45.5	26.4	< .01	48.9	44.8	37.3	< .01	45.9	43.5	.42

Self-efficacy score (percentiles)																
≤ 25^th^	33.2	26.9	28.7	35.4	< .01	38.7	32.8	26.6	.01	37.1	32.4	29.4	.09	35.2	31.8	.23
25-75^th^	31.2	34.5	31.8	30.8		30.4	31.2	32.5		28.8	33.4	33.8		29.4	32.4	
≥ 75^th^	35.6	38.6	39.4	33.8		30.9	35.9	40.9		34.2	34.5	36.8		35.4	35.8	

*Significance of overall Chi-square test of association

### Time spent in organised and non-organised activity and parent reported barriers and self-efficacy

As shown in Table 4, the number of barriers reported by parents was significantly associated with the amount of time preschool children spent undertaking organised physical activity on a daily basis (*p *< .01), with those parents reporting ≥4 barriers most often stating their child did not undertake organised activities. Over 80% of preschool children were reported to spend at least 60 mins/day doing non-organised activity, regardless of the number of barriers to activity reported by the parents. The time that preschool children spent each day in both organised and non-organised activity was not associated with parents' level of self-efficacy to influence their physical activity participation.

**Table 4:** Association between barriers, self-efficacy and time spent in organised and non-organised physical activity (mins/day)

	% participating in organised physical activity (mins/day)				% participating in non-organised physical activity (mins/day)			
	0 mins	1-59 mins	60+ mins	p*	0 mins	1-59 mins	60+ mins	p*
**Preschool group**								
Barriers (n)								
0-1	49.5	45.7	4.8	< .01	3.4	12.9	83.7	.41
2-3	64.8	29.6	5.6		1.1	13.9	85.0	
≥ 4	75.0	16.7	8.3		8.3	8.3	83.3	
Self-efficacy (percentiles)								
≤ 25^th^	59.4	38.5	2.1	.12	3.7	16.0	80.2	.21
> 25^th^-75^th^	52.3	42.7	4.9		1.2	12.5	86.3	
≥ 75^th^	51.5	41.4	7.1		2.9	11.2	85.8	
**Younger group**								
Barriers (n)								
0-1	20.5	56.1	23.4	< .01	3.4	14.8	81.8	.08
2-3	40.3	42.4	17.3		5.6	16.6	77.8	
≥ 4	63.9	23.3	12.8		13.1	19.2	67.7	
Self-efficacy (percentiles)								
≤ 25^th^	38.4	48.8	12.8	< .01	4.4	19.8	75.8	.09
> 25^th^-75^th^	26.4	57.2	16.4		3.8	16.8	79.5	
≥ 75^th^	22.1	47.5	30.4		5.1	11.4	83.5	
**Older group**								
Barriers (n)								
0-1	4.9	54.9	40.1	< .01	54.7	17.3	28.1	.52
2-3	5.9	65.9	28.2		59.9	14.8	25.8	
≥ 4	7.4	79.6	12.9		62.4	10.3	27.3	
Self-efficacy (percentiles)								
≤ 25^th^	5.3	70.3	24.5	< .01	65.0	13.5	21.5	< .01
> 25^th^-75^th^	5.1	55.5	39.3		56.1	18.0	25.9	
≥ 75^th^	5.7	48.5	45.8		47.3	18.3	34.4	

* Significance of overall chi-square test of association

Among younger children, the amount of time spent in organised, but not non-organised activity, differed significantly by the number of barriers reported by parents (*p *< .01) and the level of parental self-efficacy (*p *< .01). Parents in the highest category of self-efficacy most often reported that their children spent ≥ 60 mins/day in organised activity.

For older children, the number of barriers reported by parents was significantly associated with time spent in organised physical activity (*p *< .01), but no significant associations were found for non-organised activity. Conversely, parental self-efficacy was associated with time spent in both organised and non-organised activity (*p *< .01). The proportion of older children who reported ≥ 60 mins/day of organised or non-organised physical activity was highest among those whose parents were in the top category of self-efficacy.

### Meeting guidelines for physical activity and screen viewing in relation to parent reported barriers and self-efficacy

**Figure 1:**
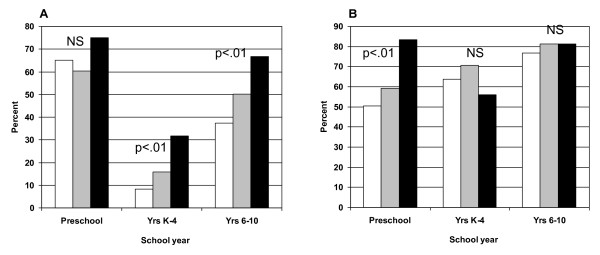
The proportion of children who do not meet guidelines for (A) physical activity or (B) screen time by the number of barriers reported by parents*. White bar 0-1 barrier. Grey bar 2-3 barriers. Black bar ≥4 barriers. *p-values derived from Chi-square test of independence

Figure 1A shows that the proportion of children not meeting physical activity guidelines was significantly higher when a greater number of barriers to physical activity were reported by parents: 8.4% (0-1 barriers) versus 31.6% (≥4 barriers) for younger children, and; 37.3% (0-1 barriers) versus 66.7% (≥4 barriers) for older children. For screen time, the proportion of preschool children not meeting the guidelines was higher when parents reported a greater number of barriers: 50.5% (0-1 barrier) compared with 83.3% (≥4 barriers) (Figure 1B). There was no significant association between the number of parent reported barriers and meeting screen time guidelines among the two other age groups.

**Figure 2:**
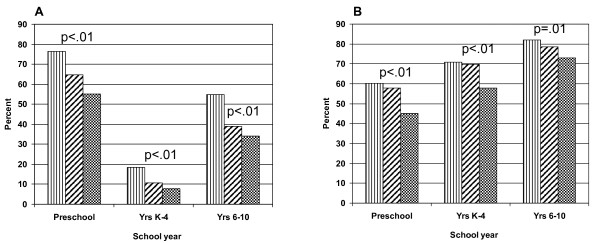
The proportion of children who do not meet guidelines for (A) physical activity or (B) screen time by the level of self-efficacy reported by parents*. Vertical lines < = 25^th ^Perc. Diagonal lines >25- < 75^th ^Perc. Black and white squares > = 75^th ^Perc. *p-values derived from Chi-square test of independence

The proportion of children not meeting physical activity recommendations also differed by level of parental self-efficacy: 76.3% (lowest category of self-efficacy) versus 55.1% (top category) among preschoolers; 18.3% versus 7.8% among younger children, and; 54.9% versus 30.7% among older children (Figure 2A). As shown in Figure 2B, the proportion of children exceeding the screen time recommendations was higher among children whose parents reported low self-efficacy than those with parents reporting high self-efficacy: 60.3% compared with 45.2% in pre-schoolers, 70.8% compared with 57.9% among younger children, and; 82.1% compared with 73% in older students.

### Multivariable analysis of factors associated with not meeting guidelines for physical activity and screen viewing

Table 5 shows that preschoolers with parents who reported high self-efficacy to influence their physical activity had lower odds of undertaking less than the recommended 3 hrs/day or more or activity. As age increased, the odds of being insufficiently active decreased (by 22% each year).

**Table 5:** Multiple logistic regression of variables associated with not meeting guidelines for physical activity and exceeding recommended screen time

Variable (reference category)	Not meeting physical activity guidelines						Exceeding screen time recommendation					
	Preschool		Younger		Older		Preschool		Younger		Older	
	Adj OR	95%CI p*	Adj OR	95%CI p*	Adj OR	95%CI p*	Adj OR	95%CI p*	Adj OR	95%CI p*	Adj OR	95%CI p*
**Child age**	‡.78	.63-.97	.95	.82-1.10	1.04	.91-1.19	†1.67	1.29-2.16	‡1.12	1.03-1.23	‡1.16	1.03-1.30
**Child sex** **(male)**	1.11	.80-1.54	†1.84	1.28-2.64	1.17	.87-1.56	.80	.58-1.12	†.64	.51-.81	†.35	.24-.51
**Region** **(urban)**	.78	.53-1.15	.73	.42-1.29	†.57	.38-.87	1.38	.97-1.96	‡.56	.36-.87	.91	.58-1.42
**Maternal education** **(Lower)**		p = .62		p = .52		p = .31		p < .01		p < .01		p = .03
Intermediate	1.18	.75-1.85	.88	.54-1.43	.71	.42-1.19	.78	.46-1.34	‡.62	.42-.93	1.18	.84-1.64
Higher	1.22	.81-1.85	.75	.44-1.27	.94	.58-1.50	†.41	.24-.72	†.43	.28-.67	.69	.48-1.02
**Household income** **(< $40,000)**		p = .42		p = .98		p = .09		p < .01		p = .81		p = .76
$40,000-$100,000	1.37	.86-2.19	.98	.51-1.86	.79	.57-1.11	.82	.50-1.33	1.19	.63-2.27	.86	.55-1.34
≥$100,000	1.25	.72-2.17	.93	.44-1.97	.43-.74	.40-.95	†.44	.26-.74	1.25	.61-2.58	.96	.57-1.62
**Total barriers** **(0-1)**		p = .03		p = .06		p < .01		p = .46		p = .06		p = .42
2-3	.69	.46-1.07	1.61	.96-2.71	‡1.63	1.14-2.32	1.16	.81-1.65	1.41	1.03-1.94	1.27	.87-1.87
≥ 4	4.25	.88-20.4	‡3.76	1.25-11.34	†3.72	1.71-8.11	3.54	.33-.37.75	1.09	.46-2.60	1.21	.48-3.06
**Self-efficacy** **(< 25^th ^%tile)**		p < .01		p = .03		p < .01		p = .01		p < .01		p < .01
> 25^th^-75^th^	.63	.38-1.05	.59	.29-1.20	†.59	.42-.84	.96	.68-1.37	.92	.67-1.26	.95	.59-1.54
≥ 75^th^	†.36	.21-.60	‡.39	.19-.79	†.45	.33-.62	‡.62	.43-.87	†.56	.39-.80	†.57	.43-.74

* Statistical significance of overall test of multi-category variable

‡ Category significantly different from reference category at .05 with Bonferonni adjustment

† Category significantly different from reference category at .01 with Bonferonni adjustment

Among young children, girls were more likely than boys to not undertake the recommended amount of physical activity of 60 mins/day or more (Table 5). The odds of not meeting the guidelines were higher among children whose parents reported ≥4 barriers, compared with those whose parents reported 0-1 barriers. Younger children whose parents reported high-efficacy were less likely to report insufficient physical activity.

Older children whose parents reported 2-3 and ≥4 barriers had higher odds of undertaking insufficient physical activity than those whose parents reported one or less barriers (Table 5). In addition, older children whose parents reported moderate or high self-efficacy, and those living in rural areas, had lower odds of reporting insufficient activity.

As shown in Table 5, age was positively associated with the likelihood of exceeding screen time guidelines within all three groups. Among the pre-school children, those with a lower likelihood of exceeding these guidelines had mothers with higher educational attainment, a family income >$100,000 and parents who reported high self-efficacy to influence their child's physical activity. Amongst younger children there were lower odds of exceeding the screen time guidelines among girls, those living in rural locations, children whose mothers had intermediate or higher educational attainment and those with parents reporting high self-efficacy. For older children, girls and those with parents reporting high self-efficacy had lower odds of exceeding the screen viewing guidelines.

## Discussion

The findings from this population-based study have shown that parent reported barriers and self-efficacy are significantly related to children's physical activity and screen time. Our analysis of the socio-demographic distribution of these factors, and their relationship with the organised and non-organised activity of children, as well as total activity and screen time, provides valuable insights for developing strategies to address physical activity and sedentariness among children of different ages.

The findings here build on past research which has found that parental support and the family environment play an important role in the physical activity participation of children [9,26]. The cost of activities and lack of opportunities in the neighbourhood were the two most common barriers to their child's physical activity that parents reported. While qualitative studies have also identified these issues as barriers that parents face [27,28], the present study has shown that these are reported more frequently at the population level than lack of time, issues with transportation or children disliking physical activity. Further, this study has revealed that cost and lack of opportunities are reported far more frequently by low-income parents, providing useful evidence for the design of physical activity strategies for low SES families. This builds on recent research in Australia [29,30] and the United Kingdom [31] indicating that household income is related to choices and level of expenditure on children's physical activities. Another insight related to structural barriers to physical activity was that lack of opportunities for physical activity and issues with transportation were more frequently reported as barriers for families in rural than urban locations, highlighting the importance of addressing these issues for rural children.

Levels of self-efficacy have been identified as a correlate of physical activity participation by school based adolescents [32], but information about parental self-efficacy to influence the physical activity of children or youth is scarce. Consistent with the barriers frequently reported by low income parents, those in the lowest category of household income most often reported lower self-efficacy. Low self-efficacy due to the expense of child physical activity was twice as prevalent among low income households, compared to those with higher incomes. Interestingly, parents of older children more frequently reported low self-efficacy to influence their child's physical activity, compared with parents of younger children, which is consistent with a recent Australian study [33]. The present findings suggest that practical communication and planning strategies targeted to assist parents of older children could be beneficial.

The multivariable analyses in this study found that both parent reported barriers and self-efficacy were associated with the likelihood of younger and older children undertaking recommended amounts of physical activity, while for preschoolers only parental self-efficacy was related to meeting physical activity guidelines. It was notable that location of residence was independently related to levels of participation among older but not younger children, with those living in rural areas less likely to be inactive. This was an unexpected finding and suggests that physical activity may play a greater part in the recreational activities of older children in the rural environment.

Consensus about the association between family SES and children's physical activity participation is yet to be reached, with some cross-sectional studies reporting lower participation levels among more disadvantaged children [34], and others not finding this relationship [35]. The present study found that, while low income parents reported more barriers and lower self efficacy to influence their children's physical activity, parental barriers and self-efficacy were related to participation levels independently of family income. This underlines the importance of addressing psychological, social and economic factors that affect the ability of parents, at all levels of SES, to enable physical activity participation by their children.

There was an opportunity in this study to examine the association between parents' reported barriers and self-efficacy and children's participation in both organised and non-organised activities. Similar to the finding reported by Heitzler et al [18] in their study of 9-13 year olds in the United States, parent reported barriers were inversely related to participation in organised physical activity but not non-organised activity. In the present study this relationship was evident, albeit in bivariate analysis, in children across a wider age range. On the other hand, parental self-efficacy was related to time spent in organised activity by younger and older children, but no association was found within preschoolers. For older children, organised sports and recreation activities not only present valuable opportunities for physical activity, but also enable the development of team work skills, leadership attributes and self-confidence [36]. If these benefits are to be available to a wide range of children this study indicates that sporting organisers need to address barriers to their programs that prospective participants may face, particularly cost, local availability of facilities and access to transport.

A growing body of research shows that sedentary behaviours, particularly screen viewing, are related to the risk of unhealthy weight gain independent of physical activity participation among primary school aged [37] and high school students [38]. There is, however, relatively little analysis of the correlates of sedentary time in children [15,39]. Here we found that parents with high self-efficacy to influence their child's physical activity were less likely to have children (across all age groups) who exceeded the guidelines for screen time. Hence, when parents report difficulty in influencing their child's physical activity the likelihood of their child exceeding screen time recommendations appears to be higher. These findings are consistent with qualitative research which has found that parents of adolescents struggle to control their child's screen time [40]. As in previous studies [41], higher levels of maternal education were also associated with limiting children's screen time, among preschool and younger children in particular. Strategies to promote understanding of the guidelines for screen time, the risk of sedentariness, and techniques for facilitating more active recreational activity need to be considered as a focus for parents with low educational attainment.

Strengths of this study were the large, random sample of parents and children who participated, and the broad age range of the children. Detailed information was collected about parents' barriers and self-efficacy, with multivariable adjustment for a range of potential confounders of the associations between these variables and child physical activity and screen time. Among the limitations of the study were its cross-sectional design, which prevents analysis of the pathways of causality between the study variables, and the use of self-report rather than objective measures of physical activity participation. Further, while the survey response rate was equivalent or superior to other population surveys of children and parents [15,18] it allowed some scope for non-response bias. For the analysis of the preschool sample the most recent guidelines for physical activity participation by Australian children up to 5 years of age were used (ie, ≥ 3 hours per day), but these recommendations have not been adopted internationally. Given the topics investigated by the surveys, respondents may have been affected by social desirability bias, however there was effort to minimise this through the use of forced choice items and self-administration [42]. Finally, the scale that was used to measure self-efficacy had good internal reliability, but its test-retest reliability has not been evaluated.

## Conclusion

This study has found that parental barriers and self-efficacy are associated with the time that children spend in both physical activity and screen viewing. Among the range of barriers investigated, cost and lack of access to facilities were most prominent, particularly for low income parents and those living in rural locations. The number of reported barriers was inversely related to time spent in organised activity by children across all age levels, and achievement of the recommended amount of total activity by younger and older children. Parent's self-efficacy was related to whether children of all ages met the recommendations for physical activity and screen viewing. These findings highlight the mix of family, economic and environmental factors that should be addressed in the design and evaluation of strategies to tackle physical inactivity and obesity among children.

## Competing interests

The authors declare that they have no competing interests.

## Authors' contributions

BS contributed to survey design and data analysis and undertook manuscript preparation. AG conducted data analysis and contributed to manuscript preparation. LH undertook survey design, facilitated data collection and contributed to data analysis and manuscript preparation. LK contributed to survey design, facilitated data collection and was involved in data analysis and manuscript preparation. LW contributed to survey design, facilitated data collection and was involved in data analysis and manuscript preparation. AM contributed to data analysis and manuscript preparation. All authors read and approved the final manuscript.

## Pre-publication history

The pre-publication history for this paper can be accessed here:

http://www.biomedcentral.com/1471-2458/10/593/prepub
